# Global Burden of Invasive Nontyphoidal *Salmonella* Disease, 2010^1^

**DOI:** 10.3201/eid2106.140999

**Published:** 2015-06

**Authors:** Trong T. Ao, Nicholas A. Feasey, Melita A. Gordon, Karen H. Keddy, Frederick J. Angulo, John A. Crump

**Affiliations:** Centers for Disease Control and Prevention, Atlanta, Georgia, USA (T.T. Ao, F.J. Angulo, J.A. Crump);; Liverpool School of Tropical Medicine, Liverpool, UK (N.A. Feasey);; Malawi–Liverpool–Wellcome Trust Clinical Research Programme, Blantyre, Malawi (N.A. Feasey, M.A. Gordon);; University of Liverpool, Liverpool (M.A. Gordon);; National Health Laboratory Service, Johannesburg, South Africa (K.H. Keddy);; University of Otago, New Zealand (J.A. Crump)

**Keywords:** bacteremia, Salmonella, incidence, global burden, invasive disease, nontyphoidal Salmonella disease, bacteria

## Abstract

Nontyphoidal *Salmonella* is a major cause of bloodstream infections worldwide, and HIV-infected persons and malaria-infected and malnourished children are at increased risk for the disease. We conducted a systematic literature review to obtain age group–specific, population-based invasive nontyphoidal *Salmonella* (iNTS) incidence data. Data were categorized by HIV and malaria prevalence and then extrapolated by using 2010 population data. The case-fatality ratio (CFR) was determined by expert opinion consensus. We estimated that 3.4 (range 2.1–6.5) million cases of iNTS disease occur annually (overall incidence 49 cases [range 30–94] per 100,000 population). Africa, where infants, young children, and young adults are most affected, had the highest incidence (227 cases [range 152–341] per 100,000 population) and number of cases (1.9 [range 1.3–2.9] million cases). An iNTS CFR of 20% yielded 681,316 (range 415,164–1,301,520) deaths annually. iNTS disease is a major cause of illness and death globally, particularly in Africa. Improved understanding of the epidemiology of iNTS is needed.

## Abstract 2

This disease is associated with approximately 3.4 million illnesses and 681,316 deaths, particularly in Africa.

Nontyphoidal *Salmonella* (NTS) disease, a major cause of diarrheal disease globally, is estimated to cause 93 million enteric infections and 155,000 diarrheal deaths each year (*1*). The Institute for Health Metrics and Evaluation estimated that enteric NTS disease was associated with 4,847,000 disability-adjusted life years lost (70 disability-adjusted life years/100,000 population) and 81,300 diarrheal deaths (1.2 deaths/100,000 population) in 2010 (*2*,*3*). However, these estimates do not include invasive NTS (iNTS) disease, which is often not associated with diarrhea. A systematic review of community-acquired bloodstream infections in Africa showed that 29% were caused by *Salmonella enterica*, and a high proportion of these infections in some parts of Africa were caused by NTS: 88% in eastern Africa, 97% in southern Africa, and 87% in western and central Africa, compared with only 1% in northern Africa (*4*). Moreover, this review identified that the 2 most common serovars causing iNTS infections were *S. enterica* serovars Typhimurium and Enteritidis, which accounted for 65.2% and 33.1%% of all NTS serotyped isolates, respectively (*4*). iNTS disease appears to be more common in some parts of Africa than in other regions of the world (*5*). Host risk factors appear to play a major role in the epidemiology of iNTS disease in Africa (*6*), where the disease is closely associated with malaria and malnutrition among infants and children (*7*–*9*) and with HIV infection among adults (*4*,*10*,*11*).

An estimate of the global and regional burden of iNTS disease is needed to inform and stimulate efforts to prevent and manage the disease. Estimation of illness and death due to iNTS disease has been limited by a scarcity of population-based surveillance data on bloodstream infections, particularly in Africa. However, the availability of high-quality country-level data on 2 major host risk factors for iNTS (i.e., HIV and malaria [*6*]) provides a basis for extrapolation of the few population-based iNTS surveillance data that are available for other areas. We sought to estimate the global incidence of iNTS disease by age and region and to calculate the number of illnesses and deaths by extrapolating credible population-based incidence data and incorporating the effects of HIV and malaria to account for differences in the population at risk among countries.

## Materials and Methods

### Systematic Literature Review for iNTS Incidence Data

We conducted a systematic review for iNTS incidence data by following guidelines of the Preferred Reporting Items for Systematic Reviews and Meta-Analyses (*12*). We used standard primary search terms (*Salmonella*, Typhi/typhoid, Paratyphi/paratyphoid, non-typhoidal, foodborne, diarrhea, Typhimurium, and Enteritidis) and standard secondary search terms (morbidity, incidence, prevalence, sequelae, mortality, mode of transmission, outbreak, invasive, bacteremia, septicemia/septicemia, bloodstream infection, invasive, CSF/cerebral spinal fluid, bone marrow, and blood culture) in the following databases: PubMed, System for Information on Grey Literature in Europe, World Health Organization library, Food and Agriculture Organization of the United Nations, and Bath Information and Data Services. Each primary search term was combined with all secondary search terms by using the AND operator. The time period for the search was January 1990–December 2012.

The types of studies included population-based incidence studies, population-based surveillance systems, and national surveillance data. We consulted with a US Centers for Disease Control and Prevention (Atlanta, GA, USA) librarian during the development of the searches. Two teams of reviewers, each led by a co-author (T.T.A. or J.A.C.) and assisted by S.H. or D.B. (see Acknowledgments), processed the citations for relevant publications in 3 stages: title, abstract, and full-text review. If there was a disagreement during any stage, a tie-breaker from the other team (T.T.A. or J.A.C.) decided if the publication was appropriate. We included foreign language articles that had at least an English translation of the title for the first stage of review. If a citation met the requirement at this stage, we looked for the English translation of the abstract for the second and subsequent stages.

### Extrapolation of Incidence to All Age Groups

**Figure 1:**
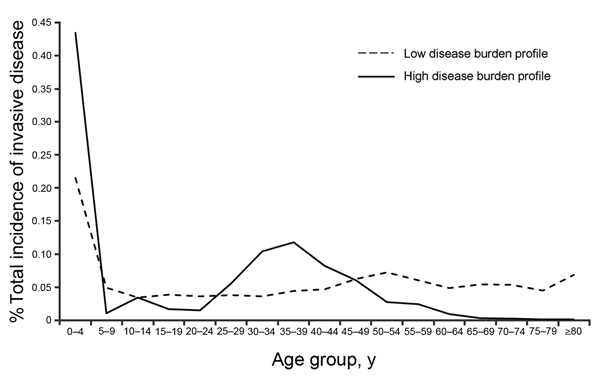
Proportion of invasive nontyphoidal *Salmonella* disease, by age group, from low-incidence settings in the United States and high-incidence settings in Malawi and South Africa 2010.

**Figure 2:**
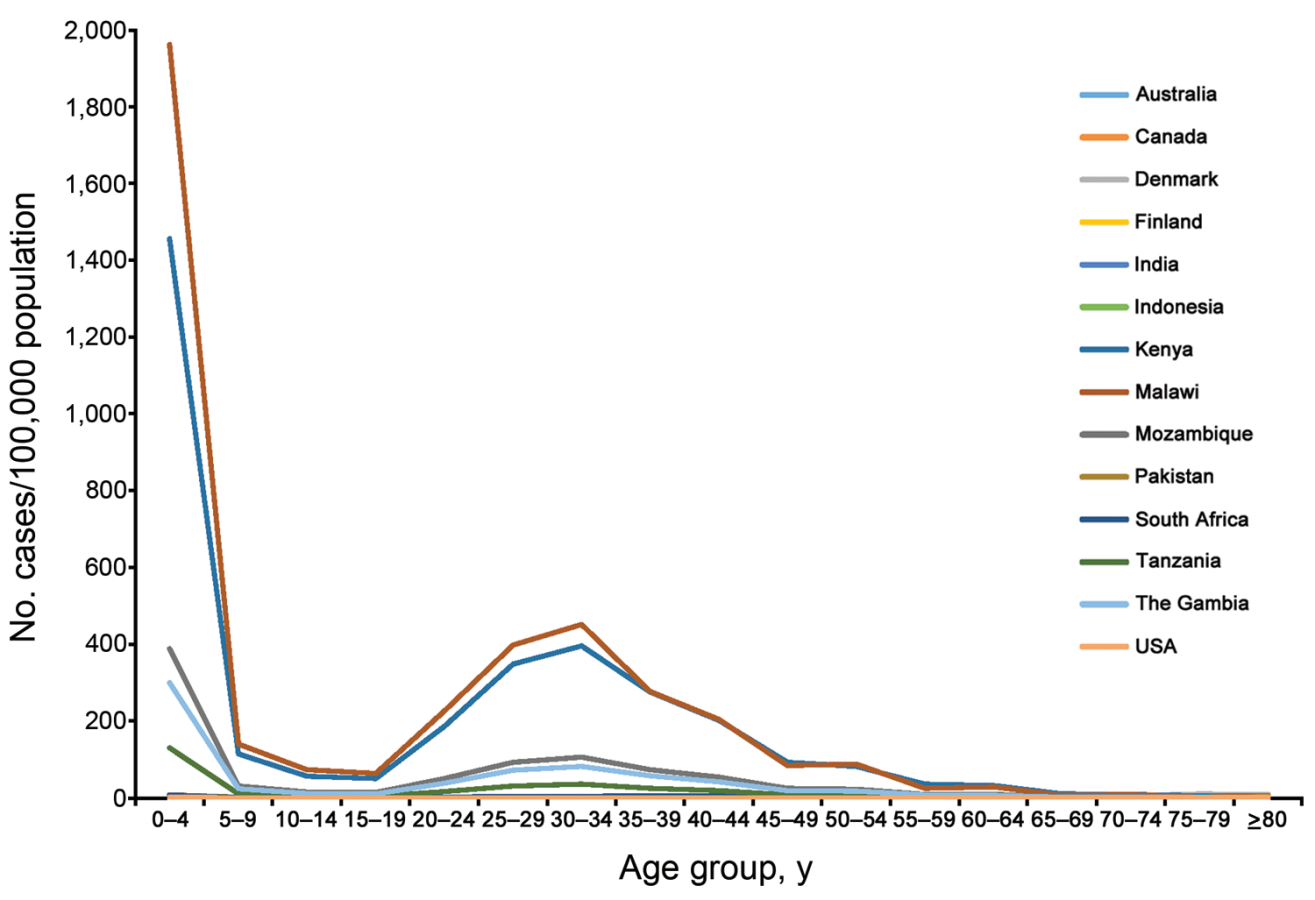
Incidence of invasive nontyphoidal *Salmonella* disease, by age group, in countries with data identified through a global systematic review of the literature 2010.

We sought to obtain incidence data for all age groups. For studies identified through the systematic review that did not have incidence data for all age groups, we created incidence profile curves to extrapolate the available incidence data to other age groups in that particular population by using proportion curves that were calculated from 2 sources that had complete case counts for each age group (Figure 1): US FoodNet (Foodborne Diseases Active Surveillance Network) data (low iNTS incidence profile) (*13*) and Malawi and South Africa surveillance data (high iNTS incidence profile) (*14*). We divided the number of cases in the available age groups by the total number of cases and used these age-specific proportions and either the low or high incidence profile, according to that country’s iNTS epidemiologic pattern, to extrapolate the incidence to the other age groups in that population. This yielded all age group iNTS incidence data for all studies identified in the systematic review (Figure 2).

### Assignment of iNTS Incidence Data to 3-Level Matrix of HIV and Malaria Prevalence

**Figure 3:**
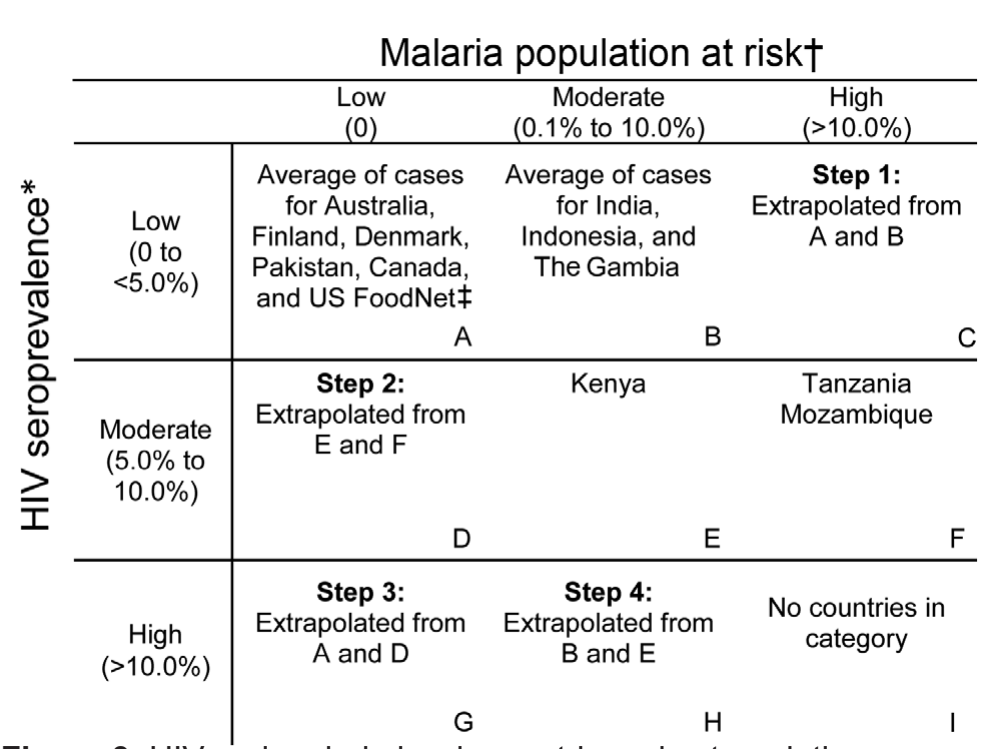
HIV and malaria burden matrix and extrapolation strategy used to create reference age-specific incidence curves for invasive nontyphoidal *Salmonella* disease. *2010 Joint United Nations Program on HIV/AIDS (UNAIDS) HIV seroprevalence (*15*); †Malaria Atlas Project population at risk (PAR) estimate, defined as the proportion of the population living in an area of known *Plasmodium falciparum* transmission (*16**, **17*); ‡US FoodNet (*13*).

**Figure 4:**
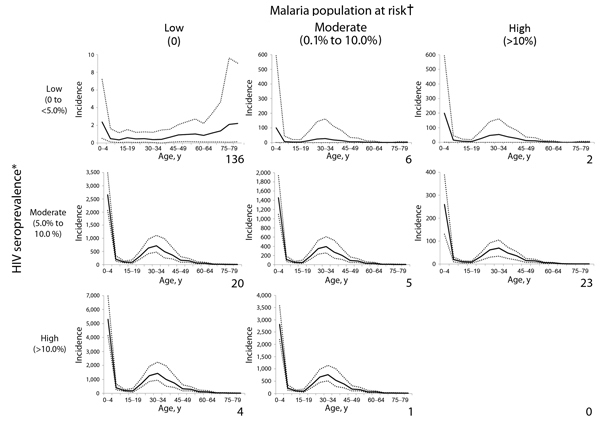
Age-specific invasive nontyphoidal *Salmonella* disease incidence (cases/100,000 population) for various HIV and malaria settings, 2010. Number of lower right corner of each chart represents the number of countries in the category. Incidence is cases/100,000 population. Solid lines on each graph represent the estimated age-specific invasive nonthyphoidal *Salmonella* (iNTS) disease incidence; dotted lines represent ranges. A country is classified into 1 of the 8 categories on the basis of national HIV seroprevalence and malaria population at risk. The age-specific iNTS incidence in that category (solid line in graph) is then applied to the country’s population data to determine the number of cases and overall incidence of iNTS for the country. *2010 Joint United Nations Program on HIV/AIDS HIV seroprevalence (*15*); †Malaria Atlas Project population at risk estimate, defined as the proportion of the population living in an area of known *Plasmodium falciparum* transmission (*16**,**17*).

We then assigned the iNTS incidence data identified from the systematic review to a 3 × 3 matrix based on the HIV seroprevalence and malaria population at risk category of the country of origin of the iNTS incidence data (Figure 3). When necessary, age incidence curves from the same matrix were also used to extrapolate data to all age groups. For HIV categorization in the matrix, we used the 2009 UNAIDS (Joint United Nations Program on HIV/AIDS) country-specific seroprevalence data (*15*) and classified countries into low (0 to <5.0%), moderate (5.0% to 10.0%), and high (>10.0%) HIV seroprevalence. For malaria categorization in the matrix, we used the Malaria Atlas Project country-specific population at risk for malaria (*16*,*17*). The population at risk is defined as the proportion of the total population living in an area of known *Plasmodium falciparum* transmission. Countries were classified as having low (0%), moderate (0.1%–10.0%), or high (>10.0%) proportions of their populations at risk for malaria. Each of the iNTS incidence data points from the systematic review (extrapolated to all age groups as described above) were assigned within the matrix, depending on the HIV and malaria epidemiology in the country source of the data. If a matrix cell had >1 data source after the iNTS incidence data were assigned, the mean incidence from all sources was calculated as the reference incidence for that matrix cell. A minimum to maximum range was also identified by using the respective values within a specific age group. If a matrix cell had only 1 incidence reference, the minimum and maximum incidences were calculated by using the median to mean ratio of cell A (Figure 3). If a cell did not have a source, we extrapolated incidence rates by using existing data, with the assumption that the middle cell was the mean of the 2 cells on either side. As shown in Figure 3, we first extrapolated cell C by assuming that cell B was the mean of cells A and C. Then we proceeded to calculate cells D, G, and H by using the same assumption. This yielded a reference all age group incidence rates for each cell in the 3 × 3 matrix (Figure 4).

### Extrapolation to All Countries Worldwide

For the last round of extrapolation, we assigned all countries worldwide to a cell in the 3 × 3 matrix according to each country’s HIV seroprevalence and malaria population at risk. We then calculated the number of iNTS cases for each country by using the reference incidence rates from the cell, according to the assignment of each country in the matrix and each country’s population stratified by age groups. We used the United Nation’s population data for 2010, medium variant, grouped by World Health Organization regions (*18*). Country classification by region, HIV, and malaria profile is available in Technical Appendix 1).

### Case-Fatality Ratio and Number of Deaths

We conducted an additional literature review to identify reports of case-fatality ratios (CFRs) due to iNTS. In addition, we solicited expert opinions in person at the 8th International Conference on Typhoid Fever and Other Invasive Salmonelloses in Dhaka, Bangladesh, 2013, and by email to develop a consensus CFR. We asked experts to provide a single value, taking into account variations by co-infections, geography, age, infection rate, strain type, and study setting (i.e., hospital based vs. community based). The consensus CFR was then applied to the estimated number of iNTS cases and the minimum and maximum range to estimate iNTS deaths and minimum and maximum range. We also varied the CFR to understand the effect of the consensus CFR on global iNTS deaths. To understand the effect of the consensus CFR, we performed a sensitivity analysis by applying the range of CFRs found in the systematic review.

## Results

**Figure 5:**
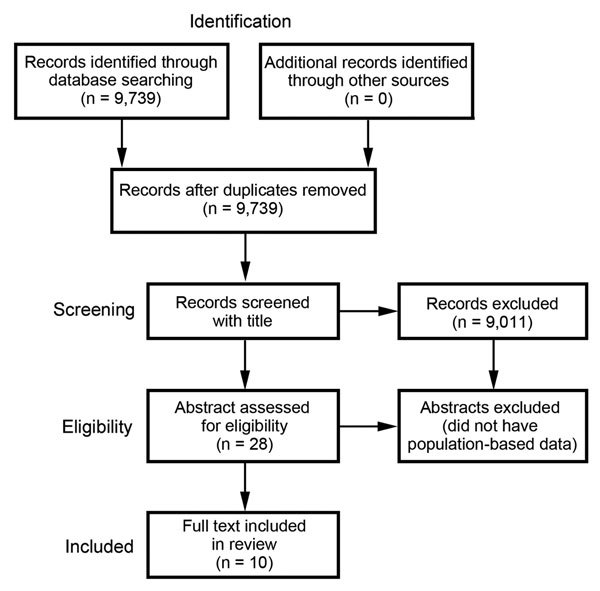
Results of a systematic review of the literature for the global burden of invasive nontyphoidal *Salmonella* disease. Reports published during January 1990–December 2012 were searched.

We identified 9,739 unique citations, 2,029 (20.8%) of which were from non–English language journals. We excluded 9,011 by title screening alone. Of the remaining 728 citations, 700 were excluded by abstract screening. Of the remaining 28 potential eligible citations with relevant abstracts, 10 were eligible for full text review (Figure 5; Table 1). 

**Table 1:** Eligible studies of incidence for iNTS from systematic literature review, 1990–2012*

Reference	Time of study	Country (city)	Median iNTS cases/100,000 population (range)
Berkley et al. (*19*)	1998–2002	Kenya (Kilifi)	8 (4–1,457)
Tabu et al. (*8*)	2006–2009	Kenya	Lwak, 232 (24–2,085); Kibera, 0 (0–260)
Nadjm et al. (*20*)	2006–2007	Tanzania	7 (0–130)
Mtove et al. (*21*)	2006–2010	Tanzania	5 (0–82)
Sigaúque et al. (*22*)	2001–2006	Mozambique	22 (1–388)
Feasey et al. (*14*)	1998–2004	Malawi, South Africa	South Africa, 1.6 (0.3–7.2); Malawi, 84 (2–1,963)
Enwere et al. (*23*)	2000–2004	The Gambia	17 (1–300)
Khan et al. (*24*)	2001–2003	China, India, Indonesia, Pakistan, Viet Nam	Pakistan, 1.6 (1.2–7.2); Indonesia, 0.2 (0.2–1.0); India, 0.05 (0.03–1.8)
Gradel et al. (*25*)	1994–2003	Denmark	1.9 (0–9.6)
Laupland et al. (*26*)	2000–2007	Finland, Australia, Denmark, Canada	Finland, 0.2 (0.1–7.6); Calgary, Canada, 0.2 (0.1–6.5); Denmark, 0.4 (0.3–1.9); Sherbrooke, Canada, 0.5 (0.4–2.2); Victoria, Canada, 0.07 (0.05–0.3); Australia, 0.1 (0.09–0.5)

*iNTS, invasive nontyphoidal Salmonella disease.

We found substantial geographic variation in age-specific incidence (Figure 2). Overall, Kenya and Malawi had higher incidence rates across all age groups compared with other countries. There was a bimodal distribution of incidence for these 2 locations, with peaks among for children <5 years of age and adults 30–35 years of age. In addition, for children <5 years of age, the incidence of disease for Kenya and Malawi was at least 3 times as high as that for other locations.

We estimate 3.4 (range 2.1–6.5) million cases of iNTS disease each year and an overall incidence of 49 (range 30–94) cases/100,000 population (Table 2). Africa has the highest incidence of iNTS disease (227 [range 152–341] cases/100,000 population) and the largest number of cases (1.9 [range 1.3–2.9]) million cases). The age group–specific incidence rate proportion profile graphs for 8 HIV and malaria scenarios suggest substantial changes in iNTS incidence rates as the HIV prevalence and proportion of population at risk for malaria increase (Figure 4). Most countries in the world (69%) belong to the low malaria and low HIV category. Among iNTS cases, 63.7% occurred in children <5 years of age globally, and 68.3% occurred in children <5 years of age in Africa. The global incidence ratio of enteric disease to invasive disease was 28:1 (range 14–45:1) (Table 2), indicating that for every iNTS case there were 28 enteric NTS cases. This ratio is highest in Asia and Oceania (3,851:1, range 1,317–23,806:1) and lowest in Africa (1:1, range 1–2:1).

**Table 2:** Global burden of invasive nontyphoidal *Salmonella* disease, 2010, compared with enteric nontyphoidal *Salmonella* disease, 2009*

Region	Enteric nontyphoidal *Salmonella*, 2009†				Invasive nontyphoidal *Salmonella*, 2010			Ratio of enteric to invasive disease (range)
	Population, thousands	No. cases	Cases/ 100,000 population		Population, thousands	No. cases (range)	Cases/100,000 population (range)	
N. Africa, MidEast	410,800	563,000	140		446,721	3,617 (660−10,483)	0.8 (0.1–2.3)	156:1 (54–853:1)
Africa	767,239	2,458,000	320		854,091	1,942,776 (1,301,399–2,910,768)	227 (152–341)	1:1 (1–2:1)
Asia, Oceania	1,628,815	53,610,000	3,280		1,693,046	13,920 (2,252–40,711)	0.8 (0.13–2.4)	3,851:1 (1,317–23,806:1)
SE Asia	2,072,274	29,839,000	1,440		2,220,248	472,263 (110,992–2,045,128)	21 (5–92)	63:1 (15–269:1)
Europe	738,071	5,065,000	690		746,372	763,191 (515375–1,179,778)	102 (69–158)	7:1 (4–10:1)
Americas	888,437	2,222,000	250		934,132	210,811 (145,145–320,732)	23 (16–34)	11:1 (7–15:1)
Global	6,511,638	93,757,000	1,140		6,894,610	3,406,579 (2,075,823–6,507,600)	49 (30–94)	28:1 (14–45:1)

*MidEast, Middle East; N. Africa, North Africa; SE Asia, Southeast Asia. †Majowicz et al. (*1*).

### CFR

A range of potential CFRs, from 3% (*27*) to 47% (*28*), was identified by the systematic review. Expert opinion identified a most likely CFR of 20%. Using a CFR of 20%, we estimated that 681,316 (range 415,165–1,301,520) deaths would result from the annual number of cases. Varying the CFR demonstrated a possible range of numbers of deaths due to iNTS from 102,197 at 3% CFR (range 62,275–195,228) to 1,703,290 at 50% CFR (range 1,037,912–3,253,800) (Table 3).

**Table 3:** Sensitivity analysis of invasive nontyphoidal *Salmonella* disease case-fatality ratio, 2010

Case-fatality ratio, %	Estimated no. annual deaths (range)
3	102,197 (62,275–195,228)
5	170,329 (103,791–325,380)
10	340,658 (207,582–650,760)
20	681,316 (415,165–1,301,520)
30	1,021,974 (622,747–1,952,280)
40	1,362,632 (830,329–2,603,040)
50	1,703,290 (1,037,912–3,253,800)

## Discussion

We estimate that 3.4 (range 2.1–6.5) million cases of iNTS disease occurred in 2010, with a CFR of 20%, for an overall incidence of 49 (range 30–94) cases/100,000 population and 681,316 (range 415,164–1,301,520) deaths. For comparison, globally there were ≈1.2 million malaria-associated deaths and 1.5 million HIV-associated deaths in 2010 (*29*). Compared with the estimated number of 93.7 million enteric NTS illnesses in 2010, the number of iNTS cases is considerably lower (*1*). However, because of the high estimated CFR for iNTS, the number of deaths resulting from this disease (681,316 deaths) is considerably higher than that estimated for enteric NTS (155,000 deaths). Furthermore, the estimated number of deaths due to typhoid fever in 2000 was 216,510 (*30*), and the estimated number for typhoid and paratyphoid fever in 2010 was 190,200 (*29*).

The highest number of iNTS cases occurred in Africa, where in 2010, almost 2 million illnesses (227 cases/100,000 population) occurred, accounting for more than half of global cases. The region with the second highest number of cases in 2010 was Europe (763,191 cases cases/100,000 population), but the number was substantially lower than that for Africa. The high number of cases in Europe is mainly driven by cases in Russia, Ukraine, and Estonia, 3 eastern European countries with large populations and relatively higher iNTS incidence profiles compared with those for countries in western Europe. Our findings, together with contemporary longitudinal descriptive literature, provide additional evidence that a largely underappreciated epidemic of iNTS has been occurring in Africa, driven in part by co-infection with HIV or malaria. Antimicrobial drug resistance emerging across the continent is likely to influence the incidence of iNTS and associated deaths (*31*,*32*).

The incidence of iNTS is highest among children and young adults in sub-Saharan Africa. These groups should be a high priority for prevention efforts, including a potential NTS vaccine (*33*). Efforts to advance vaccines against the most prevalent serotypes of NTS, *Salmonella* serovars Typhimurium and Enteritidis, should be intensified.

Estimates of illness and deaths due to iNTS both in sub-Saharan Africa and globally are likely to be inaccurate (*6*,*34*). Invasive bacterial disease surveillance is essential for identifying cases and monitoring iNTS trends, yet it is not widely available in disease-endemic areas (*35*). As demonstrated by this review, few invasive bacterial disease surveillance studies have been conducted that can provide population-based incidence data. Most published articles identified by the literature search were clinical reports or case series. Population-based incidence data are vital for future refinements of our estimates.

Sources and modes of transmission of NTS in Africa are also poorly understood. The development of nonvaccine prevention efforts will require a more in-depth understanding of the basic epidemiology of NTS on the continent (*34*,*36*). It is possible that the relative importance of transmission through food, water, and contact with animals and their environments differs from patterns observed for enteric NTS infection in industrialized nations. Furthermore, genomic studies (*37*) and integrated human and animal studies (*38*) raise the hypothesis that infected humans may be an important source of infection. There is also evidence that in South Africa iNTS is often associated with health care facilities (*39*,*40*).

Invasive NTS is a leading cause of invasive bacterial disease in Africa. This finding has a range of implications for patient management. It is vital that recommendations for empiric management of sepsis incorporate antimicrobial agents suitable for the management of iNTS. Not only are aminoglycosides inappropriate for intracellular infections, such as iNTS, but resistance to traditional first-line drugs (ampicillin, trimethoprim sulfamethoxazole, and chloramphenicol) is now common among iNTS strains in Africa (*4*). Of further concern, resistance to traditional first-line drugs among iNTS strains in Asia occurs alongside resistance to fluoroquinolones and extended-spectrum cephalosporins in some areas (*41* in Technical Appendix 2). Antimicrobial resistance appears to have played a role in the emergence and proliferation of individual NTS serovars and strains in populations at risk for infection (*31*,*32*). In sub-Saharan Africa, evidence suggests that *Salmonella* Typhimurium ST313 has developed multiple drug resistance and has adapted itself to immunosuppressed persons, particularly those living with HIV. Prevention and management of host conditions predisposing to iNTS are also likely to be key to the control of iNTS. Reductions in malaria transmission have been ecologically associated with declines in iNTS disease in some areas (*21*) (*42*,*43* in Technical Appendix 2). Declines in HIV seroprevalence and reductions in the proportion of HIV-infected persons with low CD4-positive T-lymphocyte counts by successful antiretroviral drug therapy would be anticipated to have similar effects on iNTS disease (*44*,*45* in Technical Appendix 2).

Our results demonstrate substantial differences in the ratio of enteric to invasive disease by geographic area. Whereas the incidence of enteric disease is highest in Asia and Oceania, the incidence of invasive disease is highest in Africa, where the incidence ratio of invasive to enteric disease was 1:1 (Table 2), which suggests that the magnitude of iNTS in Africa is comparable to that of enteric NTS.

Our study has several limitations. First, our rigorous systematic search for population-based incidence data in the literature resulted in a limited number of eligible sources. Despite this scarcity of eligible studies, the existing data are of high quality and, we believe, representative of the settings from where they were collected. Second, we did not consider other host risk factors, such as malnutrition and sickle cell disease. Excluding the effect of these conditions might result in underestimation of iNTS incidence rates and the number of cases and deaths. Third, we assumed that the country-level prevalence for the HIV- and malaria-infected population at risk is uniform, even though there is likely considerable subnational variation for these measures. Fourth, we did not take into account the declines in malaria worldwide in the past decade, nor did we account for changes in HIV seroprevalence and progress with provision of care and treatment globally. We used the most contemporary HIV and malaria data available. However, iNTS incidence data used in these estimations may have been collected under different HIV seroprevalence and malaria population at-risk conditions than those observed in 2010. In addition, the incidence curve for the low HIV and high malaria grouping had an artificially high peak in the 35- to 39-year-old age group, which was due to the assumption we made that, across the rows in our incidence reference grid, the middle cell is the average of the 2 cells on each side. Because we know that malaria is predominantly associated with iNTS disease among young children, we believe this artifact is a result of our assumption. However, because there were only 2 countries in this group (Comoros and Madagascar), and we believe that any potential overestimation of incidence in the 35- to 39-year-old age group in these countries will be negligible. In addition, HIV and malaria also contribute differently as risk factors in different ages in a given population. In populations with high HIV seroprevalence, the risk factors are highest among young adults and persons in younger age groups; in high malaria areas, the persons at highest risk for iNTS are those <5 years of age.

In the absence of a standard CFR for iNTS, we relied on expert opinion to estimate the most likely value. Our estimate of deaths from iNTS disease was high compared with the estimated number of deaths associated with HIV, malaria, and protein energy malnutrition. It is likely that many iNTS-associated deaths are currently counted as deaths resulting from these underlying conditions. This high estimate might have been biased by expert opinions from hospital-based studies and experiences, especially in established research programs that have better diagnostics and appropriate antimicrobial drug treatments and a higher level of care than in other settings.

Despite these limitations, we provide a baseline estimate of invasive nontyphoidal *Salmonella* disease burden globally, which is urgently needed to set the scientific and policy agenda. We hope that our estimate will be refined in the future by incorporating new population-based surveillance data, improved estimates of the CFR, and more sophisticated approaches to extrapolation and modeling. We have demonstrated that iNTS disease is a major cause of illness and death globally, particularly in Africa. Improved understanding of the epidemiology of iNTS is needed to underpin effective efforts for prevention, control, and improved patient management.

Technical Appendix 1 Country profile assignments for invasive nonthyphoidal Salmonella disease.

Technical Appendix 2 Additional references for main text.
