# Supplementary material for: Global Burden of Invasive Nontyphoidal Salmonella Disease, 2010
Source: Emerg Infect Dis. 2015 Jun;21(6):941–9. doi: 10.3201/eid2106.140999 (PMC4451910; doi:10.3201/eid2106.140999)
Supplement: Technical Appendix 2 — Additional references for main text. [file 14-0999-Techapp-s2.pdf]

Article DOI: http://dx.doi.org/10.3201/eid2106.140999

Global Burden of Invasive Nontyphoidal *Salmonella* Disease, 2010

Technical Appendix 2

References

41. Kiratisin P. Bacteraemia due to non-typhoidal *Salmonella* in Thailand: clinical and microbiological analysis. Trans R Soc Trop Med Hyg. 2008;102:384–8. PubMed http://dx.doi.org/10.1016/j.trstmh.2008.01.019

42. Mackenzie G, Ceesay SJ, Hill PC, Walther M, Bojang KA, Satoguina J, et al. A decline in the incidence of invasive non-typhoidal *Salmonella* infection in the Gambia temporally associated with a decline in malaria infection. PLoS ONE. 2010;5:e10568. PubMed http://dx.doi.org/10.1371/journal.pone.0010568

43. Scott JA, Berkley JA, Mwangi I, Ochola L, Uyoga S, Macharia A, et al. Relation between falciparum malaria and bacteraemia in Kenyan children: a population-based, case-control study and a longitudinal study. Lancet. 2011;378:1316–23. PubMed http://dx.doi.org/10.1016/S0140-6736(11)60888-X

44. Hung C-C, Hung M-N, Hseuh P-R, Chang S-Y, Chen M-Y, Hsieh S-M, et al. Risk of recurrent nontyphoid *Salmonella* bacteremia in HIV-infected patients in the era of highly active antiretroviral therapy and an increasing trend of fluoroquinolone resistance. Clin Infect Dis. 2007;45:e60–7. PubMed http://dx.doi.org/10.1086/520681

45. Larsen IK, Gradel KO, Helms M, Hornstrup MK, Jürgens G, Mens H, et al. Non-typhoidal Salmonella and Campylobacter infections among HIV-positive patients in Denmark. Scand J Infect Dis. 2011;43:3–7. PubMed http://dx.doi.org/10.3109/00365548.2010.517780
